# The role of exercise-induced peripheral factors in sleep regulation

**DOI:** 10.1016/j.molmet.2020.101096

**Published:** 2020-10-09

**Authors:** Xiao Tan, Lieve T. van Egmond, Jonathan Cedernaes, Christian Benedict

**Affiliations:** 1 Department of Neuroscience, Uppsala University, Uppsala, Sweden; 2 Department of Medical Sciences, Uppsala University, Uppsala, Sweden

**Keywords:** Exercise, Sleep, BDNF, IL-6, Irisin, PGC-a, Bmal1, Kynurenine, TNF-Alpha

## Abstract

**Background:**

Recurrently disrupted sleep is a widespread phenomenon in our society. This is worrisome as chronically impaired sleep increases the risk of numerous diseases that place a heavy burden on health services worldwide, including type 2 diabetes, obesity, depression, cardiovascular disease, and dementia. Therefore, strategies mitigating the current societal sleep crisis are needed.

**Scope of review:**

Observational and interventional studies have found that regular moderate to intensive exercise is associated with better subjective and objective sleep in humans, with and without pre-existing sleep disturbances. Here, we summarize recent findings from clinical studies in humans and animal experiments suggesting that molecules that are expressed, produced, and released by the skeletal muscle in response to exercise may contribute to the sleep-improving effects of exercise.

**Major conclusions:**

Exercise-induced skeletal muscle recruitment increases blood concentrations of signaling molecules, such as the myokine brain-derived neurotrophic factor (BDNF), which has been shown to increase the depth of sleep in animals. As reviewed herein, BDNF and other muscle-induced factors are likely to contribute to the sleep-promoting effects of exercise. Despite progress in the field, however, several fundamental questions remain. For example, one central question concerns the optimal time window for exercise to promote sleep. It is also unknown whether the production of muscle-induced peripheral factors promoting sleep is altered by acute and chronic sleep disturbances, which has become increasingly common in the modern 24/7 lifestyle.

## 1 What is sleep?

**Figure 1:**
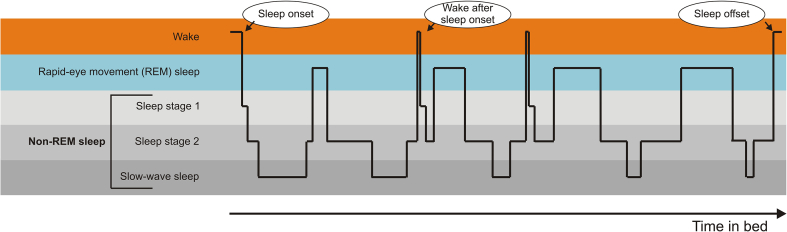
**Hypnogram depicting normal sleep in a healthy young adult.** Normal nocturnal sleep consists of 4–6 sleep cycles, each lasting around 90 min, during which non-rapid eye movement (REM) and REM sleep stage alternate. During the first half of the night, slow-wave sleep prevails, whereas REM sleep predominates the latter half of the night.

At times of high sleep propensity (i.e., at night), neurons of the hypothalamic ventrolateral preoptic nucleus release inhibitory neurotransmitters, mainly gamma-aminobutyric acid and galanin. These neurotransmitters inhibit neurons of the ascending arousal system involved in wakefulness and arousal, including histaminergic neurons and brainstem arousal regions, thereby facilitating the transition from wake to sleep [1]. By measuring eye movements, the activity of voluntary muscles, and electrical activity in the outer layer of the brain, human sleep can be divided into four distinct sleep stages ([2]; see Figure 1): sleep stage 1 (also called N1; transitional phase between wake and sleep), sleep stage 2 (also called N2; the most abundant sleep stage during sleep and hallmarked by sleep spindles and k-complexes), slow-wave sleep (SWS or N3; mainly characterized by slow, high-voltage cortical brain activity), and rapid eye movement (REM) sleep (characterized by skeletal muscle paralysis; rapid, low-voltage cortical brain activity, and intermittent REMs).

Our current understanding of how sleep is regulated comprises two highly dynamic, co-existing processes: homeostatic sleep pressure (process S) and the circadian drive for arousal (process C). Process S is dependent on wake duration and is best characterized by measuring slow-wave activity (SWA) most prominently detected over frontal cortex areas [3]. SWA is the electroencephalographic power between 0.5 and 4 Hz and mainly found during deep non-REM sleep (i.e., SWS). SWA increases as a function of preceding wake duration and decreases during sleep [4]. Process C is mainly driven by the central circadian pacemaker, the suprachiasmatic nuclei (SCN) located in the hypothalamus. The SCN regulates the timing of sleep and wakefulness and synchronizes the rhythms of peripheral metabolic oscillations. This occurs through multiple mechanisms, including rhythms in core body temperature, cortisol, melatonin, paracrine signaling, and neural circuits [5]. Light exposure leads to SCN neuronal firing by triggering electric impulses in retinal photosensitive ganglion cells. The optic nerve then transmits these electric impulses along the retinohypothalamic tract to the SCN. The SCN can be compared to an orchestra's conductor, which uses external time cues (‘Zeitgebers’), such as light, to align the sleep/wake cycle with the environmental light/dark cycle (for more details, see [6]). Apart from light, there are also non-photic Zeitgebers, such as exercise [7].

## 2 Physical exercise as a lifestyle intervention to reduce sleeplessness

Chronically curtailed and disturbed sleep are widespread in our 24/7/365 society [8]. An estimated 35–50% of adults in industrial countries report chronic insomnia symptoms, such as difficulty initiating and maintaining sleep at night [9]. Additionally, close to one billion people suffer from obstructive sleep apnea (OSA) worldwide [10]. Hallmarked by partial or total cessation of breathing during sleep, this condition can result in less restorative sleep and excessive daytime sleepiness. Finally, 20% of employees in industrialized countries have atypical work schedules. The resulting improper circadian timing of sleep due to shift work, in turn, increases the risk of sleep disturbances [11].

Chronically impaired sleep has been associated with numerous diseases that place a heavy burden on health services worldwide, including type 2 diabetes, obesity, depression, cardiovascular disease, and dementia [[12], [13], [14], [15], [16]]. Therefore, strategies mitigating the current sleep crisis are needed. In this context, promoting exercise could represent a promising lifestyle solution. Observational and interventional studies have found that regular moderate to intensive exercise is associated with better subjective and objective sleep among young, middle-aged, and older healthy subjects [[17], [18], [19]], as well as patients with sleep disorders [20]. In healthy subjects, aerobic and resistance exercise programs increase the time spent in light and deep sleep stages, enhance sleep depth, and improve sleep efficiency (i.e., the time spent asleep while in bed) [[21], [22], [23], [24], [25], [26]]. Results from a meta-analysis including 305 participants (80% female) aged over 40 years with different sleep problems suggest that regular aerobic or resistance exercise training between 10 and 16 weeks may affect sleep quality. Specifically, it was found that both types of exercise decrease the global Pittsburgh Sleep Quality Index score (PSQI, indicating better overall sleep quality, [27]). This was primarily driven by improved subjective sleep quality, shortened sleep latency, and reduced sleep medication usage [18]. In addition to its sleep-improving effects, regular exercise may also exert positive effects on sleep-disordered breathing. According to the results from a meta-analysis that enrolled 129 patients with OSA, exercise training lasting between 12 and 24 weeks resulted in a mean apnea-hypopnea index (AHI) reduction of 6.27 events/h [28]. The AHI is a clinical sleep parameter that can assess the severity of OSA [29]. Noteworthy, the exercise-induced reduction in OSA severity was achieved without a significant decrease in body weight [28].

**Figure 2:**
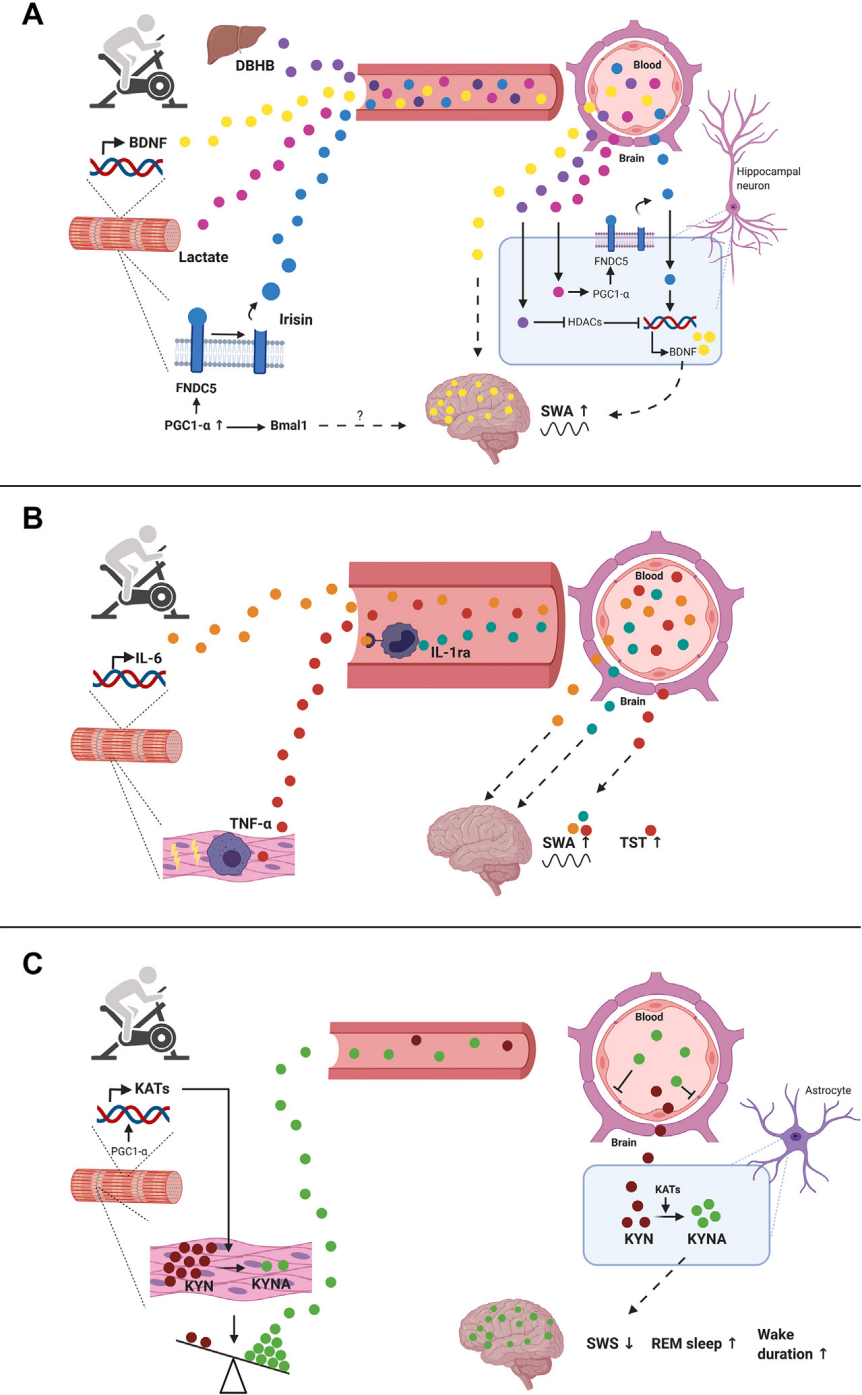
**Overview of proposed mechanisms through which exercise alters sleep in humans**. **A**) Following exercise, the *brain-derived neurotrophic factor* (*BDNF*) expression is upregulated in the skeletal muscle. Exercise also increases the hepatic production of the ketone body d-β-hydroxybutyrate (DBHB), which can increase the expression of *BDNF* in the brain (mainly the hippocampus) via inhibition of histone deacetylases (HDACs). Mediated by the protein peroxisome proliferator-activated receptor gamma coactivator 1-α (PGC1-α), exercise increases the skeletal muscle expression of *fibronectin type III domain-containing protein 5* (*FNDC5*). This membrane-bound protein can undergo proteolytic cleavage to release irisin into the blood. In turn, the myokine irisin can stimulate the expression of *BDNF* in the brain. Notably, lactate produced by the skeletal muscle following exercise can stimulate the production of irisin in the brain in a PGC-1α-dependent manner. Brain BDNF increases slow-wave activity (SWA) of non-rapid eye movement (REM) sleep, suggesting a potential role of this neurotrophin in deepening sleep. Finally, *Bmal1*, a core clock protein that is upregulated in a PGC-1α-dependent manner, may, by an unknown mechanism, influence the regulation of SWA and sleep homeostasis. **B**) In response to exercise, *interleukin-6* (*IL-6*) is expressed and released into the blood by the skeletal muscle. Circulating IL-6 stimulates macrophages to produce interleukin-1 receptor antagonist (IL-1ra). IL-6 and IL-1ra have been shown to deepen non-REM sleep (i.e., increased SWA). Microdamage in the skeletal muscle due to eccentric exercise (symbolized by the yellow flash) may stimulate the monocyte production of tumor necrosis factor-alpha (TNF-α). This cytokine may increase total sleep duration (TST) and SWA. **C**) In animals, a brain infusion of the tryptophan metabolite kynurenic acid (KYNA) reduces the time spent in slow-wave sleep (SWS) and REM sleep. Brain KYNA also increases the time awake. Due to its inability to pass the blood–brain barrier, KYNA can only be enzymatically produced by astrocytes in the brain from kynurenine (KYN). In contrast to KYNA, KYN can pass the blood–brain barrier. Exercise upregulates the skeletal muscle expression of kynurenine aminotransferases (KATs) in a PGC-1α-dependent manner. These enzymes catalyze the conversion of the KYN into KYNA. As a result, less KYN is available in the blood, with possible indirect positive effects on sleep.

Several theories exist regarding the mechanisms through which exercise may improve sleep. For instance, exercise during the day is associated with a steeper drop in core body temperature (CBT) during the evening. The decline in CBT before and during sleep represents a critical prerequisite to fall and stay asleep during the night [30]. However, less attention has been paid to the potential sleep-modulatory role of molecules expressed, produced, and released by the skeletal muscle in response to exercise. For instance, exercise increases the skeletal muscle production and secretion of brain-derived neurotrophic factor (BDNF; [31]), which is known to affect multiple tissues, including the brain [32]. Therefore, this review's objective is to provide a brief and updated overview of skeletal muscle-induced factors that may contribute to the sleep-improving effects of exercise. A summary of proposed mechanisms through which these skeletal muscle-induced molecules may alter sleep can be found in Figure 2A–C.

## 3 BDNF

BDNF is a signaling protein that is encoded by the gene *BDNF* (found in humans on chromosome 11) [33]. BDNF belongs to the neurotrophin family, regulating neurons' survival, development, and function [34,35]. Both men and women display circadian rhythms in plasma BDNF, with the peak occurring earlier in women than men (7.5 h before dim-light melatonin onset (DLMO) vs. two hours before DLMO) [36]. In addition to the circadian variation of plasma BDNF, recent studies suggest that exercise may acutely increase blood concentrations of this protein. A meta-analysis involving 910 participants (61.3% male, mean age: 42.2 years) showed that the blood concentration of BDNF increases acutely in response to aerobic but not resistance exercise training [31]. However, some evidence exists that intense resistance exercise can also elevate blood concentrations of BDNF [37,38]. As indicated by results from a more recent meta-analysis involving 1,180 participants (75.4% male; mean age: 27.9 years), the increase of BDNF in blood appears to be positively associated with exercise duration [39]. It was also observed that exercise induces a pronounced rise in blood BDNF levels in both males and females; however, the effect is seemingly more substantial in men [39]. This sex difference might be explained by lower skeletal muscle mass in women compared with men. Although exercise acutely results in elevated gene expression of *BDNF* in human skeletal muscle [40], suggesting that myocytes may drive the exercise-induced increase of plasma BDNF, this tissue is not the sole source of BDNF. For instance, both at pre-exercise rest and during a subsequent 4-h-long rowing exercise, the brain contributes 70–80% of circulating BDNF [41].

There are several hypotheses on how exercise may increase the production of BDNF in the brain. One theory is that the ketone body d-β-hydroxybutyrate (DBHB) induces *BDNF* expression in the brain (primarily hippocampus) following exercise [42]. DBHB is an energy metabolite that is increased in the liver after prolonged exercise. Circulating levels of DBHB increase in response to exercise, starvation, or absence of dietary carbohydrates [43]. DBHB is transported in the bloodstream to the brain to serve both as an energy source and signaling molecule [44]. In mice, DBHB acts as an inhibitor of class I histone deacetylases (HDACs) to activate the *BDNF* gene promoters in the brain, thus enhancing brain production of BDNF [42]. Neuronal activity is a potent stimulus eliciting the production of BDNF [45,46]. Thus, the activation of motor control-related brain areas during exercise may additionally contribute to the increased production of BDNF in the brain. It is not known whether the type and complexity of exercise and the sex of the subject alter brain BDNF; therefore, further investigation is warranted.

Supporting a mechanistic role of BDNF in sleep regulation, a study in rats showed that the larger the increase in BDNF in the cortex during wakefulness, the higher the power and incidence of SWA during the subsequent sleep period [47]. By performing unilateral cortical microinjections of BDNF in awake rats, it was further demonstrated that SWA is higher in the injected hemisphere. The same study also showed that the infusion of two different BDNF blockers during enforced wakefulness resulted in a blunted SWA response in the injected frontal cortex relative to the contralateral side during subsequent recovery sleep [36]. Altogether, these results indicate that the higher the BDNF concentration in the brain, which occurs in animals after exercise [48], the deeper is the sleep.

What are the mechanisms by which BDNF leads to an increase in rats' sleep SWA? In the brain, BDNF produces a sustained enhancement of synaptic strength [49]. As suggested by large-scale computer simulations of corticothalamic circuits under low and high synaptic strength [50,51], a local increase in the synaptic strength of corticocortical connections powerfully affects the dynamics of slow cortical oscillations. The increased coupling among connected cells also results in a more robust synchronization of single-cell slow oscillations within a network. The combination of these two effects may explain why BDNF increases SWA once it has reached the brain.

It remains unclear whether an increase in brain BDNF accounts for the sleep-deepening effects of exercise in humans. For instance, a study including a group of participants with heterogeneous sleep problems found that those with higher basal serum BDNF concentrations have lower SWS and REM sleep [52]. Of note, serum BDNF levels are increased two-to three-fold after acute exercise compared to resting conditions [53]. Thus, it remains unclear whether exercise-induced increases in blood and brain BDNF concentrations would show similar negative associations with time spent in SWS and REM sleep as described under rest conditions in [52].

## 4 Irisin

Studies have demonstrated that irisin, a 112-amino acid polypeptide hormone, is released by the skeletal muscle upon enzymatic cleavage of the membrane of fibronectin type III domain-containing protein 5 (FNDC5) in response to exercise [54]. While research suggests that irisin is involved in body weight regulation, e.g., through adipocyte browning [55], less attention has been paid to its potential role in promoting sleep upon exercise. In the acute exercise setting, irisin stimulates the expression of *BDNF* in the hippocampus of the brain [56]. As mentioned previously, BDNF injected during wakefulness into the rat brain can increase the depth of subsequent non-REM sleep [47]. However, whether an exercise-induced increase in brain irisin signaling may alter sleep directly is not known. Future mechanistic studies, e.g., using an exercise protocol in a knockdown animal model of brain irisin (as utilized in [57]) may provide valuable insights into the role of brain irisin signaling in exercise-mediated effects on sleep.

Newer research has demonstrated that irisin may partially account for the brain health-promoting effects of exercise. In an animal model of Alzheimer's disease (AD), the upregulation of irisin signaling in the brain increased neuronal synaptic strength and antagonized pathophysiological processes involved in the development and progression of AD, such as reduced binding of neurotoxic soluble amyloid-beta peptides to neurons [57]. Brain areas involved in regulating sleep and arousal, such as the SCN, can undergo neurodegeneration because of AD [58]. Thus, an exercise-induced increase in irisin may help protect these brain regions against neurodegeneration, at least when pursuing regular exercise.

## 5 Peroxisome proliferator-activated receptor gamma coactivator 1α (PGC-1α)

PGC-1α is a protein encoded by the *PPARGC1A* gene. PGC-1α increases in skeletal muscle in an exercise intensity-dependent manner [59] and can simultaneously induce transcription of a broad set of genes, most of which are involved in energy metabolism [60]. As suggested by animal studies, PGC-1α increases the production of fibronectin type III domain-containing protein 5 (FNDC5), a membrane-bound protein. Due to enzymatic processing, irisin can then be cleaved from FNDC5 and released into the circulation [55]. A recent study in mice also suggests that PGC-1α in hippocampal neurons can be stimulated by lactate to increase the production of irisin in response to exercise [61]. As mentioned in the previous section, this molecule may increase the depth of sleep by stimulating the expression of *BDNF* in the hippocampus [36].

PGC 1α also promotes the expression of the core clock gene *Bmal1* in the skeletal muscle [62]. Together with *Clock*, another core circadian transcription factor, *Bmal1* forms a heterodimer to drive the expression of *Period* (*Per*) and *Cryptochrome* (*Cry*) proteins [63]. Per and Cry proteins form a complex that translocates to the nucleus to inhibit the expression of *Bmal1* and *Clock* [64]. This ensures proper functioning of the 24-h molecular oscillator, including the skeletal muscle clock. The skeletal muscle clock's significance in muscle physiology has been addressed using genetic loss-of-function mouse models targeting the *Bmal1* gene (reviewed in [65]). For instance, using both constitutive and inducible muscle-specific *Bmal1* knockout models, insulin-stimulated glucose uptake was found to be impaired in the skeletal muscles from muscle-specific *Bmal1* knockout mice, likely due to reduced transcript and protein levels of glucose transporter 4 (*Glut4*), the insulin-dependent glucose transporter [66].

As suggested by findings from a recent animal study, skeletal muscle Bmal1 may also play a role in sleep regulation. Specifically, restoring Bmal1 in the skeletal muscle of otherwise Bmal1-deficient mice promoted the SWA rebound response to 6 h of forced wakefulness [67]. It is unclear whether direct or indirect pathways mediate the effects of skeletal muscle Bmal1 on sleep regulation. A recent study utilizing primary human skeletal myotubes found that the muscle clock plays an important role in regulating basal myokine secretion by skeletal muscle. For instance, the secretion of interleukin (IL)-6 was strongly downregulated upon skeletal myotube clock disruption [68]. Thus, it could be speculated that *BMAL1* may alter sleep through its effects on myokine production. Studies measuring the protein abundance of BMAL1 in muscle biopsies from human subjects under exercise conditions may help decipher whether the exercise-induced increase in skeletal muscle expression of *BMAL1* correlates with sleep parameters during the post-exercise night.

## 6 IL-6

The cytokine IL-6, a glycosylated protein of 21–28 kDa, is encoded by the *IL-6* gene and produced by various cells in the human body, such as monocytes and macrophages, fibroblasts, lymphocytes, myocytes, and endothelial cells [69]. IL-6 is not only involved in inflammation and infection responses but also in the regulation of metabolic, regenerative, and neural processes [70]. The cytokine can alter target cells' functions, either by binding to the membrane-bound IL-6 receptor (mIL-6r) or forming a complex with its soluble IL-6 receptor (sIL-6r) [71]. The IL-6/sIL-6r complex can act on cells that express the membrane-bound receptor glycoprotein 130 (gp130; a process named trans-signaling). Only a few cell types express mIL-6r on the cell surface (e.g., macrophages, neutrophils, some T-cells, and hepatocytes) [71]. In contrast to the mIL-6r, gp130 is ubiquitously found [71]. Under rest conditions, serum IL-6 concentrations exhibit a biphasic circadian pattern with two nadirs around 08:00 and 21:00 h and two peaks around 19:00 and 05:00 h [72]. It has also been shown that nocturnal sleep strongly enhances concentrations of sIL-6r, exceeding wake levels by 70% at the end of sleep [73]. Overall, this could indicate that the night represents a period of increased IL-6 trans-signaling capacity, with possible implications for immunity and sleep.

In addition to circadian variations of circulating concentrations of IL-6 under rest conditions, the cytokine is produced and secreted by the skeletal muscle during exercise [74]. Serum levels of IL-6 increase exponentially during exercise (up to 100-fold), and the magnitude of increase depends on exercise intensity, duration, the mass of muscle recruited, and the subject's endurance capacity [74]. Through the blood, IL-6 can reach sites behind the blood–brain barrier, but due to substantial enzymatic degradation, the blood's uptake may be limited [75]. The significant enzymatic degradation of IL-6 may also ensure that the brain's exposure to IL-6 is kept at physiologically acceptable levels. This is important as chronically elevated brain IL-6 concentrations have been tied to neuropathological changes, such as multiple sclerosis, Parkinson's disease, and Alzheimer's disease [76].

Emerging evidence from human and animal studies suggest that IL-6 may affect sleep. By utilizing the intranasal drug delivery method (a noninvasive option for delivering drugs from the nasal cavity to the brain with minimal peripheral exposure, [77]), IL-6 was shown to enhance the depth of late episodes of SWS at night (i.e., a period of increased IL-6 trans-signaling capacity, [73]), compared with placebo in 17 healthy young adults (age range 20–36 years) [78]. In a study using transgenic mice, blocking IL-6 signaling in the periphery (rather than the central nervous system) has been shown to shorten sleep duration and decrease time spent in SWS and REM sleep [79].

IL-6 may also alter sleep through interleukin-1 receptor antagonist (IL-1ra). Following exercise, IL-6 released by the skeletal muscle stimulates the secretion of IL-1ra into the bloodstream (e.g., by circulating immune cells) [80]. As suggested by a study involving 16 healthy men (mean age: 23 years), a single subcutaneous administration of IL-1ra (anakinra) before sleep increases the depth of sleep, particularly during early episodes of SWS [81].

Collectively, these findings support the hypothesis that exercise improves sleep through the IL-6 pathway.

## 7 Tumor necrosis factor α (TNF-α)

The cytokine TNF-α is a pleiotropic cytokine that constitutes a critical element in the pathogenesis of chronic inflammatory states, including rheumatoid arthritis and insulin resistance [82]. TNF-α can be directly transported from the blood to the brain [83] and induces its production in the brain via the vagus nerve [84]. Studies have shown that recombinant TNF dose-dependently enhances the depth and quantity of SWS in animal models [85]. Moreover, higher blood levels of TNF-α have been linked to higher SWA in humans with HIV [86]. The increases in non-REM sleep induced by TNF-α, depending on dose and species, persist for 4–10 or more hours [87].

TNF-α does not typically increase in response to moderate exercise in healthy subjects [80]. Moreover, the exercise-induced increase in IL-6 results in the release of soluble TNF-α receptors, which inhibit the action of TNF-α [80]. Thus, at first glance, an increase in circulating TNF-α concentration is unlikely to be a mechanism through which exercise might alter sleep. However, in contrast to moderate exercise, strenuous, prolonged exercise (e.g., marathon running) can increase the plasma concentration of TNF-α [88]. A possible explanation could be that muscle and connective tissue microtrauma caused by high volume/intensity training, with insufficient rest, can trigger the production of TNF-α by monocytes located in the recruited skeletal muscle [89]. Increased circulating TNF-α in response to vigorous exercise may explain the increase in total sleep time and SWS, as seen in athletes on four successive nights after completing a 92-kilometer road race [90]. TNF-α also increases after moderate exercise in subjects with lower exercise capacity, e.g., as shown for patients with chronic obstructive pulmonary disease [91].

## 8 Tryptophan, kynurenine, and kynurenic acid

Tryptophan is an essential amino acid critical for protein synthesis. In the brain, it serves as a precursor for sleep-regulatory signaling molecules, such as melatonin and serotonin [92]. In mammals, most of the free tryptophan is processed through the kynurenine (KYN) pathway [92]. When directed into the KYN pathway, tryptophan is converted into KYN by the enzyme indoleamine-2,3-dioxygenase (IDO) [92]. Because of its lipophilic properties, KYN readily crosses the blood–brain barrier [92]. KYN can then be converted by astrocytes into kynurenic acid (KYNA) and other active metabolites [93]. A study in adult male wild-type rats demonstrated that increased brain KYNA levels delay the onset of REM sleep, reduce total time spent in REM sleep and SWS, and increase wake duration [94]. The animals also spent more time awake during the 12-h light phase (i.e., when rodents typically rest and sleep) [94].

Endurance exercise (150 km cycling, 12 training hours per week) has been shown to increase the skeletal muscle expression of kynurenine aminotransferases (KATs) four-fold in physically active men, compared to those who had the same baseline exercise level without specific training [95]. These enzymes catalyze the conversion of KYN into the KYNA. Thus, as reviewed elsewhere [92], exercise should lower blood concentrations of KYN and concomitantly increase those of KYNA. In contrast to KYN, KYNA cannot cross the blood–brain barrier due to its hydrophilic properties [92]. Consequently, brain KYNA should drop in response to exercise, with potentially positive sleep implications, as suggested by findings described in [94].

Experiments directly measuring blood KYN levels in humans have challenged the assumption that exercise reduces blood concentrations of KYN. One study found that acute 20-minute exhaustive aerobic exercise increases serum KYN among 33 trained athletes [96]. The same study also showed that exhaustive aerobic exercise decreases the serum level of tryptophan. The drop in serum tryptophan following exhaustive exercise could result from increased uptake of this amino acid from the blood into skeletal muscles. This exercise-induced drop in tryptophan may be disadvantageous to sleep since tryptophan is required to synthesize sleep-regulatory factors, such as serotonin and melatonin, in the brain. Another study among 117 patients with mild-to-moderate depression found that a 60 min per week, 12-week exercise does not change KYN, KYNA, or the KYN/KYNA ratio in blood [97]. Given the high heterogeneity of the study population, exercise intensity, and duration of the previous studies, the possible contribution of the KYN pathway to the sleep-modulatory effects of exercise remains an important area of future research.

## 9 Conclusions and future perspectives

Exercise-induced skeletal muscle recruitment increases blood concentrations of signaling molecules, such as BDNF and IL-6, and stimulates the production of factors in the skeletal muscle, such as PGC-1α and Bmal1. As reviewed herein, these factors may promote sleep; however, evidence in humans is limited and warrants further investigation. Restorative sleep at night is essential for recovery and growth of skeletal muscles that were challenged during preceding wakefulness, e.g., through the release of growth hormone [98,99]. Thus, current evidence points toward the existence of a bidirectional relationship between sleep and exercise.

To advance our knowledge of the potential role of muscle-induced factors for the sleep-improving effects of exercise, future research should address the following questions:

1) Does the (pharmacokinetic or transgenic) inhibition of exercise-sensitive signaling pathways in the skeletal muscle alter the effects of exercise on sleep in animal models?
2) Does acute sleep loss result in a compensatory increase in muscle-induced factors promoting sleep (e.g., TNF-α) in response to exercise?
3) Is the skeletal muscle production and secretion of sleep-promoting factors in response to exercise attenuated in people with chronic sleep problems?
4) Which is the optimal time of day for exercising in order to promote sleep? Does aerobic and resistance exercise equally affect the skeletal muscle production and secretion of sleep-regulatory molecules?
5) Previous studies suggest that there are sex differences in human skeletal muscle composition and expression patterns (e.g., differences in PGC-1α expression) [100]. With this in mind, are the pathways as proposed in the present review differently affected by exercise in men and women and may differently alter sleep?

## 10 Important considerations

Other mechanisms not reviewed herein may account for variance in sleep quality and duration following exercise. For instance, high-intensity exercise increases the production of adenosine in the rat brain [101]. The accumulation of brain adenosine promotes sleep and increases SWA [102]. Reduced pain may represent an additional mechanism through which regular exercise eases sleep issues, such as insomnia. For instance, a recent meta-analysis involving 40 randomized clinical trials has shown that exercise can prevent low back pain [103], which is the most common type of chronic pain and is often associated with low sleep quality [104]. Finally, type, duration, and timing of exercise, a person's condition (disease, trained, untrained, age, gender), and genetics may alter the myokine response to exercise and are, therefore, important variables to consider.

## Funding

The authors’ work is funded by the 10.13039/501100009708Novo Nordisk Foundation (C.B., NNF19OC0056777), Skandia (C.B.), 10.13039/501100007416Swedish Brain Research Foundation (C.B., FO2020-0044), 10.13039/501100001862Swedish Research Council (C.B., 2015-03100), 10.13039/100007435Åke Wiberg Foundation (X.T., M18-0169, M19-0266), 10.13039/501100003186Fredrik and Ingrid Thuring Foundation (X.T., 2019-00488), and the 10.13039/501100006310Swedish Medical Research Society (J.C., X.T.).
